# Building towards Automated Cyberbullying Detection: A Comparative Analysis

**DOI:** 10.1155/2022/4794227

**Published:** 2022-06-25

**Authors:** Lulwah M. Al-Harigy, Hana A. Al-Nuaim, Naghmeh Moradpoor, Zhiyuan Tan

**Affiliations:** ^1^Computing, Edinburgh Napier University, Edinburgh, UK; ^2^Computer Science, King Abdulaziz University, Jeddah, Saudi Arabia

## Abstract

The increased use of social media among digitally anonymous users, sharing their thoughts and opinions, can facilitate participation and collaboration. However, this anonymity feature which gives users freedom of speech and allows them to conduct activities without being judged by others can also encourage cyberbullying and hate speech. Predators can hide their identity and reach a wide range of audience anytime and anywhere. According to the detrimental effect of cyberbullying, there is a growing need for cyberbullying detection approaches. In this survey paper, a comparative analysis of the automated cyberbullying techniques from different perspectives is discussed including data annotation, data preprocessing, and feature engineering. In addition, the importance of emojis in expressing emotions as well as their influence on sentiment classification and text comprehension leads us to discuss the role of incorporating emojis in the process of cyberbullying detection and their influence on the detection performance. Furthermore, the different domains for using self-supervised learning (SSL) as an annotation technique for cyberbullying detection are explored.

## 1. Introduction

Social media has seen exponential changes in how we network and share ideas, feelings, and information. However, ease of accessibility and anonymity welcome aggressive users and behaviors which can turn into a more serious societal issue. Cyberbullying is bullying activities that intentionally harass, insult, or abuse an individual or a group of people by repeatedly sending digital messages or comments on social media about their physical appearance, behavior, opinions, or any other subject. It can take the form of flaming, denigration, trickery, blackmailing, exclusion, outing, and cyberstalking [1, 2].

The impacts of cyberbullying on victims are as detrimental as physical bullying. The victims develop psychological burdens and suffer from emotional distress (including depression, anxiety, and loneliness) which could lead them to abuse others or to commit suicide in the worst of cases [3, 4]. Young people tend to be more vulnerable as shown in recent statistics published by various research institutions. Pew Research Centre's study reports that 59% of US teenagers were bullied or harassed in 2018 alone [5]. Approximately, 18% of children in Europe fell victim to either bullying or harassment on the Internet and mobile communications [2]. In the first two quarters of 2020, 56% of the reported cyberbullying cases in the USA occurred as a result of the increased use of Internet during COVID-19 lockdown [6].

This results in a growing need for digital cyberbullying detection to assist early intervention and prevention. Artificial intelligence (AI) techniques, particularly natural language processing (NLP), have been applied to automate cyberbullying detection by matching textual data against the identified traits of conversations on social media [7]. Formulated as a classification problem in NLP, cyberbullying detection uses sentiment analysis and document or topic classification techniques to classify messages, senders, and recipients [7]. To seek improvement to the overall efficacy of cyberbullying detection, recent research gives favour to the following technical challenges:

1. The monotonicity of NLP methods in modelling variation of natural languages results in unsophisticated detection of potential cyberbullying [8].
2. There is a lack of sufficiently large, fully annotated data for model training; annotating a large-scale cyberbullying dataset is time-consuming, labor-intensive, and error-prone as a cautious examination of multiple information sources, such as images, videos, and numerous comments, is required [9].
3. The dynamic nature of language usage and social networks means that the current guidelines for cyberbullying data annotation may not be applicable in the future [9].
4. Context-aware sentiment analysis for data annotation is proven to be critical to confirm whether a referred message is a part of an incident of online harassment directed towards an individual or a group [7].
5. The anonymous option on social media hides critical identifiable information of the actor behind a cyberbullying incident, which challenges the identification of real identity and the number of aggressors behind the incident [10].

Besides, recent research highlights that emojis along with text are widely used in communications on popular social media platforms, such as Twitter, Facebook, and Instagram, to express opinions and emotions (such as “:-)”—a smile and “:-P”—a face sticking the tongue out) to create a new form of language for social media users [11]. Emojis also help to recognise the tone of messages and the mood of their poster, and they offer a solution for the lack of non-verbal cues while only using text.

Cyberbullying detection/mitigation has attracted researchers due to its importance and potential negative impact on victims. Despite that, the literature reviews published in this area are few and do not cover all aspects because in-depth research takes time while the technology develops and changes at a fast pace and its usage increases. Salawu et al. [7] presented a survey on automated cyberbullying detection approach analysis in 2020. Their research summarized papers from 2008 to 2016 and categorized them into 4 main classes: supervised learning using ML algorithms, lexicon-based, rule-based, and mixed-initiative approaches. In addition, in their research, they covered different aspects such as the dataset creation and labelling, features conducted for cyberbullying detection, and the classifiers used by each paper. However, papers that used deep learning (DL) algorithms for cyberbullying detection and use of emojis were excluded from their analysis using self-supervised learning (SSL) for labelling datasets. Rosa et al. [12] conducted an analysis by performing quantitative systematic review of 22 studies on automatic cyberbullying detection. Elsafoury et al. [13] also presented a systematic review by reviewing the literature on automated cyberbullying detection and identifying the limitations in the available works. They also conducted experiments on the limitations they identified on the available automated cyberbullying detection and investigated their impact on the performance of cyberbullying detection. Farag et al. [14] analysed 16 studies that focused on DL with unsupervised learning (UL) and semi-supervised learning approaches that relied on unlabelled or semi-labelled datasets. They only discussed the datasets and the techniques used for cyberbullying detection. Khairy et al. [15] focused on Arabic research for detecting abusive and cyberbullying on social networks. They analysed 27 research studies regarding datasets and platform, feature engineering, and the classifiers used for detecting both abuse and cyberbullying. Krithika and Priya [16] discussed different datasets used for cyberbullying detection from different platforms. They also compared the different classifiers and feature extractions techniques used with the platform for both text and images.  Table 1 summarizes the related works mentioned above for cyberbullying detection and the domains they covered. N/A in Table 1 indicates the absence of the technique while “✓” indicates the existence of the technique.

To establish a snapshot about the state of the art in cyberbullying detection research, we conduct a comparative analysis of different detection approaches for cyberbullying on social media in this paper. It sets a focus on the approaches driven by the NLP techniques based on supervised learning (SL) and unsupervised learning (UL), as they are widely researched over other types of learning algorithms in cyberbullying detection over recent decade.

This comparative analysis will be based on the evaluation of the data annotation approaches, data preprocessing, feature extraction and engineering, and the impact of using emojis as a feature in cyberbullying detection. In addition, this paper will discuss how SSL and DL could help automate data annotation and improve classification.

To reach our objectives, different search engines and databases were used such as Google Scholar, IEEE, Springer, ACM, and others to find papers that focus on different types of cyberbullying such as abuse, offensive or hate speech, sarcasm, and irony. Seventy papers on cyberbullying detection from peer-reviewed conference proceedings and journals between 2012 and 2020 were reviewed. After filtering and then excluding papers which used non-English datasets, 45 papers were analysed for comparison.

The contributions of this paper are as follows:

- Review research on cyberbullying detection using SL, UL, and DL.
- Discuss the impact of using emojis on the performance of cyberbullying detection.
- Discuss the impact of using SSL for data annotation.

This survey will address the following issues:

- The most used ML algorithms used for cyberbullying detection in the literature (Section 3.1).
- The labelling approaches used in cyberbullying detection research (Section 3.2).
- The preprocessing approaches used for detecting cyberbullying (Section 3.3).
- The different features that can be extracted for cyberbullying detection (Section 3.4).
- The role of using emojis for detecting cyberbullying (Section 4).

This paper is organized as follows.  Section 1 will describe the related work surveys in the field of automated cyberbullying detection techniques and their limitations. The different approaches for cyberbullying detection will be analysed in Section 2 such as dataset preprocessing and labelling approaches, feature extraction, and including emojis as a feature in the detection process.  Section 3 will discuss the effect of including emojis on cyberbullying detection performance. Section 4 will explain the self-supervised learning (SSL) approach and how to use it for labelling the datasets. Sections 7 and 8 will contain the conclusion and the future work of this research, respectively.

## 2. Cyberbullying Detection Approaches

### 2.1. Machine Learning Paradigm

Most of the research reviewed in this paper such as Ptacek et al. [17]; Nand et al. [18], Fariaset al. [19], and Bharti et al. [20] used supervised learning for cyberbullying detection where the datasets need to be labelled. To avoid manual labelling of the huge datasets, Capua et al. [21] used unsupervised learning (UL) by applying a clustering algorithm using growing hierarchical self-organizing map (GHSOM) networks which used a hierarchical structure of multiple layers of many independent self-organizing maps (SOMs). Romsaiyud et al. [22] also used an UL approach to detect cyberbullying by feeding the texts into a cluster and a discriminant analysis stage to identify abusive texts and later clustering them as polite messages and abusive messages. The contents of the messages are identified based on a crime pattern and the normalized documents using the K-means clustering technique.

They also used Naïve Bayes to build a predictive model to classify the abusive texts into one of the eight predefined categories which included activity approach, communicative, desensitization, compliment, isolation, personal information, reframing, and relationship.

Cheng et al. [23] proposed a model called XBully for cyberbullying detection that incorporates multi-modal network embedding through exploiting social media information such as image information, user information, and network information using machine learning algorithms.

#### 2.1.1. Deep Learning (DL) Approaches

Deep learning (DL) is considered part of a broader family of machine learning algorithms based on learning data representations which consists of various techniques such as deep neural network (DNN), recursive NN, recurrent NN, convolutional NN, and deep belief networks [24]. Although there is a growing body of research on the topic of DL, very few studies have applied DL techniques for cyberbullying detection [25].

The main arguments for the use of DL in cyberbullying detection are as follows [25, 26]:

- Its ability in discovering complicated structures in high-dimensional data.
- Learning the most significant features automatically from a general learning procedure.
- It requires very little engineering by hand like machine learning algorithms.

In addition, DL has algorithms that can be used in cyberbullying detection using emojis such as CNN which may provide higher performance results on extracting contextual features for classification tasks in images [27].

Zhao and Mao [28] developed a text representation model called semantic-enhanced marginalized denoising autoencoder (smSDA) using one of the DL methods called stacked denoising autoencoder (SDA). SDA contained a semantic dropout, designed based on domain knowledge and word embedding, and sparsity constraints. Their proposed model solved the issue of the robust and discriminative numerical representation learning of text messages to reduce the ambiguity of these messages on social media as these messages are short and contain a lot of misspelling and informal languages. Research by Agrawal and Awekar [27] supported the effectiveness of using DL algorithms in detecting cyberbullying for textual words by comparing the performance of using deep neural networks with the use of machine learning in multiple social media platforms. For their experiment, they used four different ML algorithms and proposed four different DL models that differed only in the neural architecture layer while being identical in the rest of the layer. They used four different DL algorithms for the neural architecture layer: CNN, LSTM, BLSTM, and BLSTM with attention.

Rosa et al. [25] also used three DL algorithms to implement three architectures: CNN as detailed by Kim [29]; C-LSTM as detailed by Zhou et al. [30]; and Mixed CNN-LSTM-DNN from Ghosh and Veale [31]. Cai et al. [32] proposed a multi-modal hierarchical fusion model for detecting sarcasm in Twitter using the text, image, and attribute features. Subramanian et al. [33] used DL for detecting sarcasm in social networks using word embeddings (Word2Vec) and emoji embeddings (emoji2Vec). In addition, their model focused on the sarcasm part of the sentence by using attention layer. Cheng et al. [9] proposed an unsupervised learning approach (UL) for cyberbullying detection using clustering methods based on deep neural network (DNN). Their model contained two main components: a representation learning network and a multi-task learning network.

Mozafari et al. [34] and Paul and Saha [35] used the BERT model to detect cyberbullying and hate speech in social networks. Mozafari et al. [34] used a pretrained BERT model with transfer learning to enhance hate speech detection by using fine-tuning strategies to examine the effect of different embedding layers of BERT in hate speech detection. They leveraged syntactical and contextual information of all transformers of BERT for detecting hate speech.

Pavlopoulos et al. [36] used two models: perspective API for detecting offensive content and BERT for identifying the offensive categories. For perspective API, they used a toxicity model which is a CNN based on GLOVE word embeddings trained on millions of user comments. For BERT, they used a base version of BERT with 12 transformer layers to detect both a masked word from its left and right context and the next sentence. Paul and Saha [35] fine-tuned the BERT model for detecting cyberbullying. However, the BERT model comes with huge computational cost as it consists of a huge number of parameters. Tripathy et al. [37] used fine-tuned ALBERT model for cyberbullying detection by using ALBERT-large, a larger pretraining corpus over the BERT-Base that Mozafari et al. [34] implemented. Elsafoury et al. [13] conducted experiments using BERT for cyberbullying detection in comparison to the DL models used in the literature. The results of their experiments found that using BERT improved the detection, and they found that there is lack of research on the use of BERT for cyberbullying detection.

### 2.2. Dataset Labelling and Evaluation Approaches

#### 2.2.1. Data Labelling Approaches

Data labelling is the process in which sentences, messages, or posts in the dataset are tagged into several categories based on the definition given for each category. This process is very important for supervised learning algorithms as the classifiers need annotated data to train themselves based on the labels.

**Figure 1:**
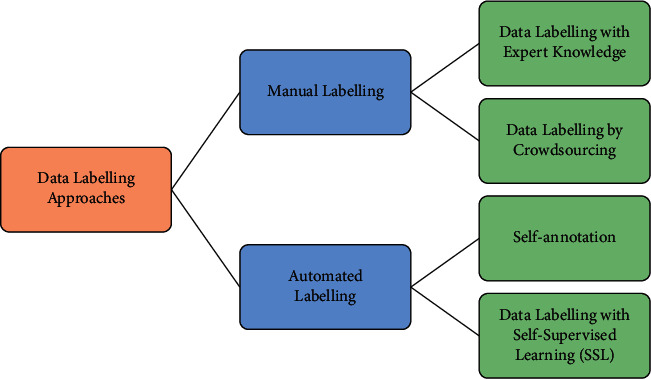
A taxonomy of data labelling approaches.

Different approaches have been used in recent research for data labelling. The taxonomy shown in Figure 1 classifies various labelling approaches into one of the two categories: manual labelling and automated labelling, to reflect the involvement of human expertise.

*(1) Manual Labelling*. Manual labelling approaches use knowledge from domain experts and/or crowdsourcing in data annotation. The recent advantage and applications of manual labelling are introduced below. This is the most used type of annotation techniques found in the literature. Using experts for annotation is desirable for the research to ensure high-quality annotated data which is a barrier that crowdsourcing services will need to overcome. Using crowdsourcing, in contrast, is relatively low in cost and saves time for data labelling [7]. Manual data labelling has two main types using expert knowledge or crowdsourcing as follows:

(a) Data labelling with expert knowledge: human domain experts play a key role in labelling, and their expertise has a direct influence on the quality of labelled data and affects the modelling accuracy indirectly. Many researchers used experts in the field of cyberbullying definition to annotate their datasets. The types of experts involved in the labelling process are as follows.
  - Trained annotator: Van Hee et al. [38] used linguistic students and second-language speakers of English to annotate their dataset. Patro et al. [39] used the dataset released by Joshi et al. [40] that was also manually annotated by three linguistic experts who had linguistic annotation experience for tasks such as sentiment analysis, word sense disambiguation, and other related works. Fortunatus et al. [4] also manually annotated their Facebook dataset using two native English speakers, but they evaluated their results using Cohen's kappa.
  - Social media participant: Mishra et al. [41], Romsaiyud et al. [22], Agrawal and Awekar [27], and Ortega-Bueno et al. [42] used participants to annotate their datasets.
  - Third-party expert: Potha and Maragoudakis [43] manually annotated the questions of the predators using a numeric class label. Ptacek et al. [17], Wallace et al. [44], Nand et al. [18], Zhang et al. [26], and Zhao and Mao [28] used two to three expert annotators to manually annotate their data. Van Hee et al. [38] also used experts for annotating their tweets, but they adopted an annotation guideline scheme developed by Van Hee et al. [45].
  - Researchers themselves: Mishra et al. [46] and Mozafari et al. [34] used a dataset released by Waseem and Hovy [47] and annotated by its authors themselves as racism, sexism, and neither.
(b) Data labelling with crowdsourcing: data labelling crowdsourcing is obtaining data annotations from contributors with mixed knowledge and experience via crowdsourcing platforms, such as Amazon Mechanical Turk, CrowdFlower, and Eight Flags on the Internet. Crowdsourcing service was defined by John et al. [48] as involving “organizations using information technology to engage crowds comprised of groups and individuals for the purpose of completing tasks, solving problems, or generating ideas.” The two main advantages of crowdsourcing are the ubiquity and low cost of network connectivity and the speed and low cost of large-scale data processing [49]. The most used crowdsourcing websites in this research's analysed papers are as follows.
  - Amazon Mechanical Turk: Reynoldset al. [50], Capua et al. [21], and Rosa et al. [25] used experts from Amazon's Mechanical Turk to annotate their datasets for cyberbullying. Paul and Saha [35] used three datasets that were labelled manually for cyberbullying also, either using experts or Mechanical Turk crowdsourcing.
  - CrowdFlower: different data categorizations were used in the papers included for analysis in this research. Farias et al. [19], Hosseinmardi et al. [51], and Rafiq et al. [52] follow the same categorization, where a message is labelled as either cyberbullying or cyberaggression. Hosseinmardi et al. [51] used five contributors (i.e., the labellers) and provided them with the images/videos to be annotated and their associated comments as well as the written definitions of cyberbullying and cyberaggression.
  - A slight retermed categorization is used in Davidson et al. [53] and a follow-up research by Tripathy et al. [37], where the provided tweets to be annotated are labelled as one of the three categories: hate speech, offensive, or neither.
  - Samghabadi et al. [54] used three datasets, and one of them was released by Wulczyn et al. [55] and labelled using the CrowdFlower website. The second dataset released by Samghabad et al. [56] was also labelled using the CrowdFlower website, but they used three contributors providing them simple annotation guidelines for some positive and negative examples to ease their task. The third was the shared task of “Detecting Insults in Social Commentary.”
  - Figure Eight: Zampieri et al. [57] proposed three-level hierarchical annotation schema for abusive language detection. Their schema encompasses three general categories: (i) offensive language detection to distinguish between whether the language is offensive (OFF) or (NOT); (ii) categorization of offensive language to distinguish its type as targeted insult (UNT) and untargeted insult (TIN); and (iii) offensive language target identification to distinguish its target as individual (IND), group (GRP), and other (OTH). They provided their schema to annotators from the *Figure Eight* platform. The authors hired only the qualified annotators, who must have experience with the platform and passed a selection assessment. They used two annotators for each instance and requested a third annotator in case of disagreement and then took a majority vote.

*(2) Automated Labelling*. An automated labelling approach requires no human intervention and uses either self-annotation techniques or self-supervised learning (SSL). The types of automated labelling are as follows:

- Data labelling by self-annotation: the self-annotation (or self-tagging) approach is the most commonly used automated annotation approach in the surveyed literature, by searching for a word or hashtag within posts, through which a message is annotated based on its presence.
- The presence of #sarcasm or #sarcastic, considered in Amir et al. [58] for example, suggests a label “sarcasm” to the tagged tweets associated with it. A similar approach is employed in Cai et al. [32] to annotate a newly collected set of English tweets for multi-modal sarcasm detection. The tweets containing a picture and some special hashtags (e.g., #sarcasm) are positive examples for sarcasm, and those with images but no such hashtags are negative for sarcasm.
- Attempting to enhance the reliability of data labelling, a hybrid approach is used in Farias et al. [19], where self-tagging is used together with crowdsourcing for data annotation. Hashtags, including #irony, #sarcasm, and #sarcastic, are used to flag and tag for a post as irony. The work also concludes the efficacy of the hybrid approach in comparison with the self-tagging and crowdsourcing annotation. Six datasets were involved in this study.
- Apart from hashtags, sentiment keywords, such as love, amazing, good, hate, sad, happy, bad, hurt, awesome, excited, nice, great, and sick, can also help with self-annotation. Bharti et al. [20] demonstrated the use of such sentiment keywords together with hashtags to label data for training sarcasm post detectors. The list of sentiment keywords was learned using parsing-based algorithm for lexicon generation (PBALN) algorithm.
- Zhang et al. [59] conducted a comparative study to better understand the efficacy of self-annotation. Seven publicly available datasets were chosen in the study that were annotated using either a self-tagging or a manual annotation approach. A manually annotated dataset was chosen from the seven datasets and reannotated using hashtags. The data annotated with the two strategies were used to train their DL-based sarcasm detection model separately in order to compare the performance for sarcasm detection between these two strategies. The results did show that the model built with the self-tagged data outperformed the manually tagged one. The authors believed this was the result from the contribution of self-tagging, which is more efficient in labelling and capable of supplying more annotated tweets for training than manual annotation.
- Another interesting recent research by Samghabadi et al. [54] suggested that transferring knowledge learned from a dataset to annotate another set of data does not always result in desirable outcomes even though both sets of data share the same format. The empirical results showed a degradation in labelling when applying a classifier, trained using ask.fm dataset, on their Curious Cat dataset. They found that the quality of the automatic labelling might be affected due to the differences between these two platforms (Curious Cat and ask.fm). Although these two platforms had the same format, the ask.me dataset was created based on profane words while they found numerous sexual posts in Curious Cat.
- Data labelling with SSL: Yann LeCun, the Turning Award Winner in AAI conference 2020, described self-supervised learning (SSL) as “the machine predicts any parts of its input for any observed part.” SSL is similar to UL as both techniques do not involve manually labelled datasets. However, UL concentrates on clustering, grouping, and dimensionality reduction, while SSL aims at recovering and drawing conclusions for regression and classification tasks which is still in the paradigm of supervised settings [60]. A detailed discussion of SSL can be found Section 6.

From our point of view, using data labelling with an expert knowledge approach for annotating the data either using students, participants, or the researchers themselves is more efficient as the researchers can choose the annotators with specific background knowledge, e.g., annotating data for bullying or non-bullying, and give the annotators the exact definition of cyberbullying that they want them to follow, but it might take time. However, using crowdsourcing for labelling the dataset could be faster, but the annotation quality is lower than using experts. On the other hand, using the self-annotation approach is low cost and fast, but using hashtags or bad words to annotate data does not always give accurate results of the performance because it does not take into account the context around the word as sometimes it is used in a context different from its meaning. The different types of annotation used in the surveyed literature are summarized in Table 2.

#### 2.2.2. Dataset Utilization

Different types of data collected from social network platforms can be utilized by researchers for cyberbullying detection. Some of those researchers used one type of platform for their research and the others used more than one, which can be used to support that their proposed model is able to work on different social network platforms. Therefore, the following is how datasets are utilized using a single dataset or multiple datasets.

*(1) Using a Single Dataset*. Using a single dataset depends on the social network platform such as

- *Twitter*. The most popular and most widely used platform is Twitter with publicly available datasets released by researchers. Ptacek et al. [17] and Rajadesingan et al. [61] retrieved their own datasets using Twitter to train their models for sarcasm detection. Ptacek et al. [17] collected Czech and English Twitter datasets while Rajadesingan et al. [61] collected English tweets only with the keywords #sarcasm and #not at the end. Rajadesingan et al.'s [61] dataset was used for sarcasm detection by other research such as Zhang et al. [26]. Some researchers used a subset of publicly available datasets such as Amir et al. [58] who used a subset of Bamman and Smith [68] Twitter corpus and replicated their experimental setup.
- Van Hee et al. [38] collected only English tweets from Twitter using irony hashtags such as #irony, #sarcasm, and #not. Their main goal was to develop a set of annotation guidelines for identifying specific aspects and forms of irony to be able to model irony in text, without relying on additional information provided by the author such as hashtag. Nand et al. [18] created their own Twitter dataset by using TAGS archiving tool to download 2500 public tweets from the Twitter community. TAGS is a free Google Sheet template which can be used to set up and run automated collection of search results from Twitter. They retrieved the tweets using recommended bullying keywords in psychology literature from Ortony et al. [69]; Cortis and Handschuh [70]; Squicciarini et al. [71]; Browne [72]; and Ybarra [73] such as nerd, gay, loser, freak, emo, whale, pig, and so on. Mishra et al. [46] conducted their experiment using a Twitter dataset of Waseem and Hovy [47] annotated for abuse under the categories: racism, sexism, or none. Patro et al. [39] used a dataset which had two types of sarcastic textbook snippets and tweets. Drishya et al. [63] exploited an Instagram dataset to detect cyberbullying from both text and images.
- Singh et al. [74] used a Twitter dataset released by Van Hee et al. [62] which had two tasks: binary and multi-class classification. Zampieri et al. [57] retrieved their dataset from Twitter using API searching for offensive keywords using a list that included these keywords such as “he is,” “she is,” and “you are.” They excluded some of these keywords with the lowest concentration of offensive content to keep the distribution of offensive tweets at around 30% of the dataset such as “they are.” They used a hierarchical annotation scheme split into three levels to distinguish between whether the language is offensive or not (Subtask A: OFF, NOT), its type (Subtask B: TIN, UNT), and its target (Subtask C: IND, GRP). Their dataset was used by many researchers such as Pavlopoulos et al. [36] and Hettiarachchi and Ranasinghe [64]. Hettiarachchi and Ranasinghe [64] used the dataset to detect cyberbullying using capsule network architecture which incorporated emojis.
- *Wikipedia*. Iwendi et al. [2] used an English dataset from Wikipedia that was released by Wulczyn et al. [55]. The dataset was binary classified by labelling the comments with 0 for neutral and 1 for bullying.
- *Perverted-Justice*. Potha and Maragoudakis [43] utilized a dataset of real-world conversations from Perverted-Justice, an American organization, as pairs of questions and answers between the cyber predator and the victim and modelled each set of predator's questions as a time series. The site consisted of volunteers who posed as minors by choosing the age range for the decoys from 10 to 15 and waiting for adults to approach them.
- *Reddit*. Wallace et al. [44] used a subset of the reddit irony dataset which was collected and labelled by Wallace et al. [75] which contained 1825 annotated comments from 876 progressive and 949 conservative subreddits.
- Fortunatus et al. [4] used Facebook, specifically Melania Trump's Facebook post comments, as she received a lot of attention from citizens of the USA as the wife of the President at the time. They extracted the dataset using a Python scraping program.
- *Curious Cat*. The Curious Cat website is a semi-anonymous, question answering social media platform like ask.fm. Samghabadi et al. [54] collected their fourth dataset using Curious Cat website.

*(2) Using Multiple Datasets*. Zhao and Mao [28] crawled their datasets using two public real-world datasets: (1) a Twitter dataset: contained tweets crawled by collecting tweets that start with “bull” including “bully,” “bullied,” and “bullying” and then the tweets were manually labelled as bullying or non-bullying. (2) a MySpace dataset: crawled from MySpace groups and each group contained different posts by different users which can be regarded as a conversation about one topic. Each data sample was defined as a window of 10 sequential posts. Romsaiyud et al. [22] also crawled their datasets and used two different datasets one from Perverted-Justice as training datasets and another Twitter dataset from Stanford University as testing datasets.

Mishra et al. [41] used two publicly available datasets which contained eye-movement information: dataset 1 released by Mishra et al. [76] and dataset 2 used by Joshi et al. [77] where both datasets were binary classification with positive or negative emotions. Cheng et al. [23] also used two publicly available datasets from two different platforms, an Instagram dataset released and collected by Hosseinmardi et al. [51] and a Vine dataset released by Rafiq et al. [52]. Mozafari et al. [34] used two Twitter datasets for their BERT model provided by Waseem and Hovy [47] and Davidson et al. [53]. The first dataset was annotated as racism, sexism, and neither while the second dataset was annotated as hate, offensive, and neither.

Subramanian et al. [33] utilized two different datasets from different platforms, Facebook and Twitter, collected from sarcastic pages such as “sarcasmLOL” and “sarcasmBro” from Facebook and tweets with hashtags “sarcasm” and “sarcastic” from Twitter. Cheng et al. [9] used Instagram dataset collected and released by Hosseinmardi et al. [51] and Vine dataset collected and released by Rafiq et al. [52]. Rezvani et al. [67] also used Instagram dataset from Hosseinmardi et al. [51] and a Twitter dataset collected and labelled by Sui [78], respectively. González et al. [65] conducted a dataset for their experiments for irony detection on English and Spanish datasets by using the corpus of the Irony Detection on Spanish released by Ortega-Bueno et al. [42] and the English Tweets released by Van Hee et al. [62].

Capua et al. [21] used three datasets from three different social network platforms: a Formspring.me dataset taken from Kontostathis et al. [79], a YouTube dataset taken from Dadvar and Jong [80], and a Twitter dataset taken from Sanchez and Kumar [81]. Agrawal and Awekar [27] targeted different types of social media networks, and each dataset represented a different topic of cyberbullying, for example, Formspring presented bully word datasets; Twitter presented racism and sexism word dataset; and a Wikipedia talk pages presented personal attack word dataset. Rosa et al. [25] trained three different text representations through the word2vec model from three different dataset sources: Google News, Twitter, and Formspring, using two versions of each dataset: balanced and unbalanced. Samghabadi et al. [54] used three publicly available datasets from ask.fm, Wikipedia used by Samghabad et al. [56] and Wulczyn et al. [55], and the shared task of “Detecting Insults in Social Commentary,” respectively. In addition, they collected their own dataset from the Curious Cat website which is a semi-anonymous, question answering social media platform like ask.fm. Paul and Saha [35] used three publicly available datasets from three different platforms, Twitter dataset released by Waseem and Hovy [47], Wikipedia dataset released by Wulczyn et al. [55], and Formspring dataset released by Reynolds et al. [50]. The Twitter dataset had three classes, racism, sexism, and none, the Wikipedia dataset had two classes, attack and not attack, and the Formspring dataset had two classes, bullying and non-bullying.

Potamias et al. [66] conducted their experiment on four publicly available datasets Irony/SemVal-2018-Task 3.A, Reddit SARC2.0 politics, Riloff sarcastic, and SemEval-2015 Task 11.

Farias et al. [19] used six Twitter datasets for irony detection used by other researchers TwReyes2013, TwIronyBarbieri2014, TwSarcasmBarbieri2014, TwPtacek2014, TwMohammad2015, and TwRiloff2013.

### 2.3. Data Preprocessing

Preprocessing is an important step to refine the dataset and reduce the noise and the size of the data by removing unwanted information and preparing the dataset for feature extraction and training the classifiers for the classification process. Reducing the noise by removing the unwanted words and symbols increases the performance of the models which are only trained on important words. Data preprocessing can have many steps such as removing stop words and punctuations, tokenization, stemming, lemmatization, removing URLs, hashtags and retweets (mentions), and lower case letters, and removing emojis/emoticons or replacing them with their descriptions or Unicode. The following are the most widely used preprocessing steps in the literature.

#### 2.3.1. Replacing Mentions (@), Hyperlinks (URLs), Hashtags (#), and Retweets (RT)

It is the most commonly used preprocessing technique which removes mentions, URL links, hashtags, and retweets from the posts. Most of the researchers removed these unwanted symbols to clean the datasets of noise as in Potha and Maragoudakis [43], Ptacek et al. [17], Rajadesingan et al. [61], Van Hee et al. [38], Nand et al. [18], Bharti et al. [20], and Felbo et al. [82]. Rosa et al. [25] used the Formspring dataset which is based on question and answer, so they need to remove “Q” and “A” markers as a preprocessing step. Some of the researchers kept the hashtag texts as they might include important text, for example, Singh et al. [74] removed the hashtag symbols (“#”) and expanded the hashtag texts such as #PickANewSong which became pick a new song.

#### 2.3.2. Stop Words/Punctuations

Removing stop words and punctuations is also a common step for preprocessing that aims to eliminate words and marks that appear to be of little value to reduce noise in the data such as is, are, am, a, an, the, he, she, them, do not, and so on. Punctuations are markers that can be used in writing to separate sentences, phrases, and clauses to clarify or emphasize the sentence meaning such as full stop “.,” comma “,,” question mark “?,” exclamation mark “!,” semi-colon “;,” colon “:,” and others. Most researchers removed stop words and punctuation as they considered them as noisy words such as Zhao and Mao [28], Mishra et al. [46], Singh et al. [74], Subramanian et al. [33], Fortunatus et al. [4], Gupta et al. [83], Iwendi et al. [2], and others.

#### 2.3.3. Tokenization

It is a process for breaking sentences and phrases into a sequence of sentences or words called tokens. These tokens are then converted to numerical representation to be used and trained by the model. For example,

  Sent = “This product is great. It makes working a lot easier”
  Sentence level tokenization would be
 
*S* = [“This product is great,” “it makes working a lot easier”]
  Word level tokenization would be
 
*W* = [“This,” “product,” “is,” “great,” “it,” “makes,” “working,” “a,” “lot,” “easier”].

Tokenization was used by many researchers such as Potha and Maragoudakis [43], Ptacek et al. [17], Zhao and Mao [28], Singh et al. [74], González et al. [65], and Hakak et al. [84].

#### 2.3.4. Stemming

These are algorithms which cut off the end or the beginning of the word, taking into account a list of common prefixes and suffixes that can be found in an inflected word. The words “studied” and “studies” will be converted to “stud” by truncating the suffixes. Many research studies used stemming such as Potha and Maragoudakis [43], Ptacek et al. [17], Capua et al. [21], Drishya et al. [63], and Hakak et al. [84].

#### 2.3.5. Lemmatization

It converts each word to its root such as converting “studying” and “studies” to “study” and converting “is,” “are,” and “am” to “be.” Stemming and lemmatization are sometimes used alongside each other such as in Iwendi et al. [2].

The most used preprocessing steps are replacing @, #, URL, and RT and removing stop words, even though using DL and pretrained models such as BERT do not need removing them since removing them could modify the meaning of the sentence.

Table 3 demonstrates the different preprocessing steps conducted by the researchers in this survey. “✓” in the table demonstrates the existence of the technique.

### 2.4. Feature Engineering

Feature extraction is an essential step for detecting cyberbullying where the most important features are determined. In DL, feature engineering is an implicit step as the features are automatically extracted by DL models while for ML, we need to manually extract and engineer them. Choosing the most significant features from a large number of raw data reduces the dimensionality and the amount of redundant data and also reduces the processing time. The following section will be divided into three subsections according to the source of the feature such as text or image and the other subsection including the research that used other types of features.

#### 2.4.1. Text Features

Ptacek et al. [17] used various N-gram features such as character n-gram, and skip-bigram, pattern of the word frequency, part-of-speech feature (POS), and other features such as number of positive and negative emoticons and number of punctuations. Rajadesingan et al. [61] identified different forms of sarcasm and categorized them as one or combination of different forms. They divided the features into sets depending on the different forms of sarcasm and analysed features to find which features had the most contribution in detecting sarcasm in tweets. They found 10 most important features such as the percentage of emoticons, adjectives, past words with sentiment score, positive to negative sentiment transitions made by the user and capitalized hashtags in the tweet, etc. They found that all these features were derived from all sarcasm forms that they determine. Capua et al. [21] used about 20 hand-crafted features as input layer by utilizing four different groups of features in their proposed model for detecting cyberbullying using the UL approach. The features were syntactic features, semantic features, sentiment features, and social features.

Farias et al. [19] used two types of features: structural and affective features. The structural features included the length of words, emoticons, discourse markers, part of speech, semantic similarity, and added eight features such as the length in characters, colon, exclamation, question, the number of uppercase characters, hashtags, mention frequency, and retweets. For affective features, they followed Farias et al. [85] model by using affective information as features to represent ironic tweets such as dictionary of affect in language (DAL) and sentiment lexicons, sentiment-related features, and emotional categories.

Van Hee et al. [38] implemented four feature groups which trained a binary classifier based on each of these features:

- For lexical features, they used BoW, unigrams, bigrams, trigrams, and four grams with a set of numeric and binary features.
- For syntactic features, they integrated part-of-speech, binary feature indicating a “clash” between verb tenses in the tweet, and four features (one binary and three numeric) indicating the presence of named entities in a tweet that used to indicate (i) the number of named entities in the text, (ii) the number, and (iii) frequency of tokens that are part of a named entity.
- For sentiment lexicon features, they used six sentiment lexicon features based on publicly available sentiment lexicons, and for each lexicon, they derived five numeric features and one binary feature.
- For semantic information, they used word embedding cluster features generated with Word2Vec.

Nand et al. [18] used filtered and unfiltered psychometric and word features. Zhao and Mao [28] solved the data sparsity which affected the generalization of the testing messages. Their proposed model used the BoW model as an input to represent a document in a textual corpus using a vector of real numbers that indicated the frequency of the words in the document. Bharti et al. [20] used part-of-speech (POS) tagging as a feature. Mishra et al. [46] used community-based profiling features of the tweet's author and investigated the effectiveness of incorporating the authors' profile when examining their tweets considering the properties of the authors' community information. The researchers also created an undirected unlabelled community graph for authors who created the tweets in the dataset, where the nodes and the edges denoted the authors and the connections between them, respectively. The authors who neither follow any other author nor were followed by any were denoted as nodes with no edges and indicated as solitary authors. Their results showed that inclusion of the user's profile and community information improved the performance of their proposed method for detecting abuse.

Patro et al. [39] used socio-linguistic features along with text features to detect sarcasm targets in sarcastic texts with their DL model. They identified various socio-linguistic features that differentiate the target text from the rest of the snippet/tweet. They found that adding additional socio-linguistic features into their DL framework improved performance for both snippets and tweets. Samghabadi et al. [54] proposed a model that predicted the abuse by capturing the local and sequential information from the text using a neural network. They proposed another mechanism called emotion-aware attention (EA) that incorporated emotional information from the text to find the offensive words inside the text. Cheng et al. [9] exploited multi-modal features such as text, network, user, and time features. They used representation learning network models using a hierarchical attention network (HAN) for textual features and a graph autoencoder (GAE) for user and network features and used the multi-modal representations (e.g., text, user, and social network) as an input to estimate the bullying likelihood using a time-informed Gaussian mixture model (GMM).

Fortunatus et al. [4] combined textual features together for detecting cyberbullying including emojis and emoticons. They processed the cleaned text using different scores to produce a series of scores forwarded to be classified. They used a combination of scores for the sentences to classify an input such as text aggression score, text positive word score, text sentiment score, emoticon sentiment score, and emoji sentiment score. The text aggression score was calculated to find the score for each word in the sentence using a swearword lexicon which was taken from different sources such as https://noswearing.com, https://bannedwordlist.com, https://cs.cmu.edu, https://rsdb.org, and bad words list from Engman's thesis [86]. They focused on features such as including modifier handling, but check, and least check.

#### 2.4.2. Image Features

Zhong et al. [87] focused on detecting cyberbullying on Instagram using DL in commentaries following shared images on Instagram. They investigated leveraging different features extracted from images in detecting cyberbullying in posted comments such as image-specific, text features extracted from comments and image captions, topics determined from image captions, and outputs of a pretrained convolutional neural network applied to image pixels. They used DL algorithms for image clustering aiming to group similar images together which could correspond to similar bullying signature and then extract image features using a pretrained CNN. Mishra et al. [41] proposed a model based on convolutional neural network (CNN) that extracted cognitive features from the eye-movement and gaze data of human readers reading the text. They used these cognitive features along with textual features for the tasks to classify the input text for sentiment polarity and sarcasm detection. Cai et al. [32] took advantage of three types of features: text, image, and attribute features, and used them in their multi-modal hierarchical fusion model for detecting sarcasm in Twitter. They used the image attribute features to initialize a bi-directional LSTM network (Bi-LSTM) for extracting the text features. Cheng et al. [23] proposed a model for cyberbullying detection that incorporates multi-modal social information such as image, user profile, time, and location. They used five modalities extracted from Instagram: user, image (including number of shares, likes, and label of the image), profile (including number of followers and total number of comments and likes), timestamp of posting, and the textual information.

Drishya et al. [63] extracted features from both text and image in their Instagram dataset. They used the bag of words model (BoW) to extract the features by associating each word with its frequency of occurrences. Each text is represented as a feature vector that contained binary attributes for each and every word that occurred in that message. Image, feature vectors, and feature map were the three inputs to the CNN classifier. Rezvani et al. [67] proposed a model that exploited three types of features for cyberbullying detection: metadata features, image features, and enrichment features. Metadata features were extracted from the metadata information associated with the social contents such as number of followers and following, number of likes, average of reactions and replies, and number of mentions. Images were labelled using ImageNet model and built image extraction features which produced a Boolean occurrence vector with labels in each element of the vector. Enrichment feature is a Boolean vector containing the occurrences of the profanity words took from the knowledge base of Google's standard profanity word list. They preprocessed the list to produce a smaller list and then looked up through their dataset to find the appearance of the profane words from the list.

#### 2.4.3. Others

Wallace et al. [44] exploited different types of features by using the subreddit type as a feature for each comment (progressive or conservative; atheism or Christianity) and combined it with the sentiment of the comment (negative, positive, or neutral) and the extracted noun phrases from the comments. Amir et al. [58] proposed a neural network model that explicitly learned and exploited user embeddings in conjunction with features derived from utterances. Zhang et al. [26] also used the automatic features from neural networks and compared them with the discrete manual feature models for detecting sarcasm. Their proposed model contained two main components: a local component to extract features from the target tweet itself and a contextual component to extract contextual features from the history of the target tweet, which can reflect the tendency of the author in using irony or sarcasm. They used bi-directional gated recurrent neural network (GRNN) to capture syntactic and semantic information over tweets locally and a pooling neural network to extract contextual features automatically from history tweets.

Using DL layers to extract features automatically was conducted by Hettiarachchi and Ranasinghe [64] and Potamias et al. [66].

## 3. Role of Emojis in Cyberbullying Detection

People use several communication forms for both written and spoken languages to express their feelings with their facial expressions, mimics, and gestures. Such forms can be replaced by emojis for digital communication in human and machine interconnected applications [88]. There are a variety of emojis available that cover every basic concept and mood which make a strong connection between the emojis and their semantical meaning [89]. Including emojis in communication between social media users can better help them express their emotions or certain opinions, which cannot be expressed by voice or body language through textual communication. Emojis were first released in 2010 and consist of a set of various symbols ranging from cartoon facial expressions to figures such as flags and sports [11]. The growth of using emojis started between 2011 and 2013 when major mobile phones began to support emoji keyboards to their devices such as Apple and Android [90].

Short text length, the use of pseudowords like # hashtags or @ mentions, and even metadata such as user information or geolocalization are essential components of social media messages [91]. Moreover, the use of emojis, people, and scenes is becoming increasingly important for fully modelling the underlying semantics of a social media message [91]. Emojis are extensively used, not only as sentiment carriers or boosters, but also more importantly, to express ideas about a myriad of topics, e.g., mood (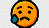), food (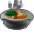), or sports (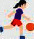) [91]. However, using emojis can help users to combine special tones through social media especially for those without an intuitive understanding of the language [92].

The Oxford Dictionary defines an emoji as “a small digital image or icon used to express an idea or emotion” (e.g., 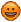, 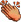, 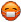) [74]. Emojis are interesting because they succinctly encode meaning that otherwise would require more than one word to convey (e.g., grinning face, clapping hands, and face with medical mask for the emojis above) [74].

In many cases, sentences without using emojis can lead to a different meaning and can be understood to be positive since they have positive words such as “WOW,” “beautiful,”…, and so on [33]. For example, using emojis in a sarcastic sentence like “WOW!! it is beautiful 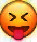
” is an indication for bullying because of the use of a negative emoji 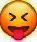 although it contains positive words like “WOW” and “beautiful” [33]. Removing emojis from such sentences through the cyberbullying detection process will change the polarity of the sentences by considering them inoffensive which would lead to wrong results.

The use of text by users of social media, such as Twitter, Facebook, and Instagram, is still the most popular form of communication between users although images and videos are also used [33].

Few researchers incorporated emojis or emoticons for detecting cyberbullying. Hettiarachchi and Ranasinghe [64] integrated emojis for detecting offensive content using separate embedding in addition to character embedding emoji2vec consisting of pretrained embeddings for all Unicode emojis using their descriptions in the Unicode emoji standard. Samghabadi et al. [54] proposed an emotion-aware attention mechanism that found the most important words in the text by incorporating the emotional information from the text. They created an emoji vector using a DeepMoji model to capture the emotion from the text and preparing an emoji vector by tokenizing it to sentences and made an emoji vector as a binary representation by assigning 1 to the five most probable emojis and 0 to the others. They found that angry emojis were highly correlated with the offensive class, while happy and love faces appeared more frequently in the neutral class. Singh et al. [74] also incorporated emojis for cyberbullying detection and sentiment analysis by replacing all emojis with their textual descriptions.

Subramanian et al. [33] integrated emojis with text for sarcasm detection by using neural network to learn the connection between emojis and text. They found the top 20 emojis used in Twitter and the top 5 emojis used in Facebook in sarcasm based on their count of occurrences in the comments. The types of emojis used across the comments in Twitter and Facebook datasets were analysed to explore the rank of the top emojis used. The face with stuck out tongue emoji is the most used on Facebook and third on Twitter while a face with tears of joy emoji is the most used on Twitter. However, the face with tears of joy emoji is being increasingly used in both sarcastic and non-sarcastic comments across platforms.

Fortunatus et al. [4] incorporated emojis and emoticons for cyberbullying detection by calculating scores for text, emojis, and emoticons. Text scores were calculated for each sentence while emoji and emoticon scores were calculated once for a single input.

Gupta et al. [83] proposed a system to detect sarcasm considering text and emoticons using artificial neural network (ANN) as a classifier. They identified the polarities for both the sarcasm text and the emoticons as positive and negative and trained them using ANN. If the polarity of the text was positive, the positive emoticons were uploaded; otherwise, the negative emoticons were uploaded; then, they trained them again to classify whether the text is considered sarcasm or not.

Subramanian et al. [33] conducted different experiments by training their proposed model for detecting sarcasm using Word2Vec and emoji2Vec. They found that training their model using emoji2Vec only performed better than the performance when using Word2Vec only while concatenating both Word2Vec and emoji2Vec performed the highest. Hettiarachchi and Ranasinghe [64] integrated emoji knowledge to detect type and target of offensive posts in social media using capsule network which consisted of four layers: embedding layer, feature extraction layer, capsule layer, and dense layer. The performance of their model incorporating emojis had higher performance for detecting offensive content than the model of Zampieri et al. [57] which did not include emojis.

## 4. Self-Supervised Learning (SSL) Approach

Supervised learning (SL) is one of the machine learning approaches which can learn from labelled datasets while the unsupervised learning (UL) approach such as K-means and hierarchical clustering is used to make clusters for unlabelled dataset as the dataset collected from online social media does not come with labels [93]. Using supervised learning to label datasets using human or automatic annotations is expensive and time-consuming while using an UL approach results in less efficiency [93]. The lack of annotations of the huge amount of data pushed researchers to find alternative approaches that can annotate them, and this is where self-supervised methods play a vital role in fuelling the progress of DL without the need for expensive annotations and learning feature representations where data provide supervision [94].

Although supervised approaches have shown promising results for detecting cyberbullying, they suffer from two major limitations: (1) annotating large-scale datasets of cyberbullying which require cautious examination of multiple information sources such as images, videos, and numerous comments is time-consuming, labor-intensive, and error-prone; (2) due to the dynamic nature of language usage and social networks, current guidelines for labelling a sentence as cyberbullying may not be effective in the future [9].

Self-supervised learning (SSL) is a hybrid approach as no manual label is involved, which is the same as UL, and the feature extraction ability is rapidly approaching the SL method [60]. Moreover, UL focuses on detecting specific data patterns, such as clustering, while SSL aims at recovering, which is still an example of supervised settings [60]. SSL has the following advantages:

- It is used to avoid time-consuming and expensive data annotations by learning features from large-scale unlabelled images or videos without using any human annotations [95].
- It can be used for a textual dataset by learning useful knowledge representations and the sentiments exhibited by the users without having to use an annotated and labelled dataset [93, 96].
- It narrows the gap between supervised and unsupervised learning techniques for learning meaningful representations by reducing the requirement of labelled activity data, effectively [97].
- It can be used in both the image and text features to alleviate the need for large, labelled data by deriving labels for the significantly more unlabelled data [98].
- It allows the models to be modified or trained entirely from scratch [93].

SSL is mostly used by researchers for sentiment analysis either using textual or visual content.

### 4.1. Textual Data

Lin et al. [99] proposed an SSL method for sentiment classification used to reduce the load of manually labelling data. They proposed a multi-lingual sentiment analysis framework to estimate the sentiment polarity of reviews with no manually labelled corpus with the key sentences automatically extracted from unlabelled data. They compared the performance of their proposed framework with UL and SL baseline frameworks from the literature. Their experimental results showed that their proposed framework with SSL had a higher performance for estimating the sentiment polarity than all the UL baselines and supervised baselines based on the Naive Bayesian algorithm.

Lan et al. [100] introduced a self-supervised loss for sentence-order prediction (SOP) to improve the performance of ALBERT as SOP focused on inter-sentence coherence and was designed to address the ineffectiveness of the next sentence prediction (NSP) loss proposed in the original BERT.

Kokatnoor and Krishnan [93] used a machine learning approach to identify and detect the different categories of anomalies for a Twitter dataset on user's opinions on demonetization policy in India. They labelled the textual dataset with three labels: positive, negative, and neutral, using SSL and an UL approach (K-means clustering algorithm), resulting in three clusters. Their experimental results showed that using the SSL approach had higher accuracy for detecting anomalies when compared to a conventional UL approach.

### 4.2. Visual Data

Gomez et al. [101] used SSL for self-supervised feature learning of visual features by mining a large-scale corpus of multi-modal (text and image) documents. They trained a CNN to learn visual features from images and predict the semantic context in which a particular image is more probable to appear as a clarification. The researchers performed many experiments to show the quality of the visual features learned by their text topic predictor (TextTopicNet).

Bai et al. [102] used SSL for learning image features by predicting anatomical positions automatically defined by cardiac chamber view planes to train a cardiac MR image segmentation network.

Huang et al. [103] used SSL to address the problem of labelling video datasets that required a huge number of human annotators. SSL was used in their proposed model motion from static images (MoSI) to train video models by learning representations from either video or image datasets. For video datasets, the source images need to be first sampled from the videos by sampling one frame out of each video randomly as the source image, while for image datasets, no frame-sampling step is required.

## 5. Conclusion

According to the literature, there is a growing body of cyberbullying detection research; however, there is a need for more research to enhance the area in terms of using new pretrained models such as BERT, ALBERT, and other models.

Supervised learning (SL) is the most popular approach that is used for cyberbullying detection where all the datasets were labelled either manually or automated. In this survey paper, we discussed and analysed the different methods that were used to detect cyberbullying, using either ML or DL, starting from data evaluation and labelling which is the most important step. A manually annotating approach is the most used approach among the researchers especially using crowdsourcing platforms probably because it is fast, especially when used with large-scale datasets. We also discussed the most important steps used for data preprocessing and cleaning, and the most used preprocessing step is removing @, #, RT, and stop words. In addition, self-supervised learning (SSL) can be used for automatically annotating data without the need for human intervention which can save time. Furthermore, from the literature, emojis play a big role in the detection process of cyberbullying through social networks. Taking emojis into account can be a method for increasing the performance of the model as they have the ability to change the sentiment and context of the intended meaning of the sentence [104–108].

## 6. Future Research Direction

From our review of the research in the field of cyberbullying detection, we found other interesting issues that need to be investigated further.

### 6.1. SSL for Cyberbullying Dataset Annotation

The vast majority of the papers included in our survey paper used manual approaches for data annotation while the minority used automated approaches. Using manual approaches such as crowdsourcing has a relatively high cost but saves time while using participants such as experts, students, or the researchers themselves for a lower cost, but it is time-consuming. On the other hand, using an automated approach by using self-tagging such as using hashtags or specific words is low in cost and saves time, but it does not always give accurate results as it does not take into account the context of the sentences. Using hashtags or bullying words in the posts is not always an indicator of bullying and it depends on the context of the conversation. Recent advances in data annotation using SSL for labelling cyberbullying datasets has highlighted their effect in annotating textual and visual data. It is worth using SSL for labelling large-scale cyberbullying datasets by learning the features from the data without using any human intervention. Also, it is interesting to investigate the efficiency of using SSL for labelling datasets while keeping emojis by using them as symbols inside the posts or replacing them with their Unicode.

### 6.2. Incorporating Emojis in Cyberbullying Detection

Using emojis is very popular among social media users in normal conversations to express their feelings, emotions, and opinions which is why some social networks allow only a limited number of words in each post. Since emojis have proven efficient in the sentiment analysis domains, it is, therefore, important to incorporate them in cyberbullying detection as they play a role in determining the context of the sentence while ignoring them can change the whole meaning of the sentence. In other words, removing emoji from a sentence considered offensive and intimidating sometimes can lead to changing the sentiment of the sentence from negative to positive. We need a deeper investigation into the meaning of emojis, how they are used for bullying on social media, and the most used emoji symbols for such a purpose.

### 6.3. Bullying Victim's Emotional Behavior

Bullying has a dangerous impact on the emotional feelings and behavior of the victims as they suffer from negative emotions about their lives such as depression, anxiety, and loneliness, which can lead them to suicide, in extreme cases. The reaction of each victim differs from one individual to another, as some of them may disappear from the social networks or shut down their accounts, and others may change their name or status, while others may write negative words describing their emotional state. There is a lack of cyberbullying detection research using sentiment analysis for victims' emotional behavior by analysing the victims' emotions and attitudes to hopefully save and support the victims.

## Figures and Tables

**Table 1:** Summary of the related work surveys.

Research	ML approach	Dataset	Labelling approach	Feature engineering	Preprocessing technique	Classifiers	Using emojis	Using SSL
[7, 12]	ML, SL	✓	✓	✓	✓	✓	N/A	N/A
[14]	DL with UL and semi-supervised learning	✓	N/A	N/A	N/A	✓	N/A	N/A
[15]	ML and DL with SL and UL	✓	N/A	✓	N/A	✓	N/A	N/A
[16]	ML and DL	✓	✓	✓	N/A	✓	N/A	N/A
[13]	ML and DL	✓	N/A	✓	N/A	✓	N/A	N/A
This survey	ML	✓	✓	✓	✓	✓	✓	✓

**Table 2:** Types of data labelling used in the recent research.

Research	Platform	No. of datasets	Language	Type of cyberbullying	Type of annotation	Annotation method/platform
Reynolds et al. [50]	Formspring.me	One	English	Cyberbullying	Manual	Amazon Mechanical Turk
Potha and Maragoudakis [43]	Perverted-Justice	One	English	Cyberbullying	Manual	Experts
Ptacek et al. [39]	Twitter	One	English and Czech	Sarcasm	Manual	Experts
Rafiq, et al. [52]	Vine	One	English	Cyberbullying	Manual	CrowdFlower
Rajadesingan et al. [61]	Twitter	One	English	Sarcasm	Automated	Self-annotating using hashtags
Wallace et al. [44]	Reddit	One	English	Irony	Manual	Experts
Amir et al. [58]	Twitter	One	English	Sarcasm	Automated	Self-annotating using hashtags
Capua et al. [21]	Formspring.me, YouTube, and Twitter	Three	English	Cyberbullying	Manual	Amazon Mechanical Turk
Farias et al. [19]	Twitter	Six	English	Irony	Manual and automated	Crowdsourcing and self-annotating using hashtags
Van Hee et al. [38]	Twitter	One	English	Irony	Manual	Participants
Hosseinmardi, et al. [51]	Instagram	One	English	Cyberbullying	Manual	Crowdsourcing
Nand et al. [18]	Twitter	One	English	Cyberbullying	Manual	Experts
Zhang et al. [20]	Twitter	One	English	Sarcasm	Manual	Experts
Waseem and Hovy [47]	Twitter	One	English	Hate speech	Manual	Authors themselves
Zhao and Mao [28]	Twitter and MySpace	Two	English	Cyberbullying	Manual	Experts
Bharti et al. [20]	Twitter	One	English	Sarcasm	Automated	Self-annotating using hashtags
Davidson et al. [53]	Twitter	One	English	Hate speech	Manual	CrowdFlower
Mishra et al. [41]	Twitter + snippets with eye movement	Two	English	Sarcasm	Manual	Participants
Romsaiyud et al. [22]	Twitter and Perverted-Justice	Two	English	Cyberbullying	Manual	Participants
Samghabad et al. [56]	Ask.fm	One	English	Nastiness	Manual	CrowdFlower
Wulczyn et al. [55]	Wikipedia	One	English	Personal attack	Manual	CrowdFlower
Agrawal and Awekar [27]	Formspring, Twitter, and Wikipedia	Three	English	Cyberbullying	Manual	Participants
Van Hee et al. [62]	Twitter	One	English	Irony	Manual	Linguistic students and second-language speakers of English
Mishra et al. [46]	Twitter	One	English	Abuse	Manual	Authors themselves
Rosa et al. [25]	Google News, Twitter, and Formspring	Three	English	Cyberbullying	Manual	Amazon Mechanical Turk
Cai et al. [32]	Twitter	One	English	Sarcasm	Automated	Self-annotating using hashtags
Cheng et al. [23]	Instagram and Vine	Two	English	Cyberbullying	Manual	Crowdsourcing
Drishya et al. [63]	Instagram	One	English	Cyberbullying	Not mentioned	
Mozafari et al. [34]	Twitter	Two	English	Hate speech	Manual	Authors themselves
Patro et al. [39]	Book snippets and tweets	One	English	Sarcasm	Manual	Experts
Samghabadi et al. [54]	Ask.fm, Wikipedia, Kaggle, and Curious Cat	Four	English	Abuse	Manual and automated	Using CrowdFlower and pretraining
Hettiarachchi and Ranasinghe [64]	Twitter	One	English	Offense	Followed Zampieri et al.'s [57] approach	
Ortega-Bueno, et al. [42]	Twitter	One	Spanish	Irony	Manual	Participants
Subramanian et al. [33]	Facebook and Twitter	Two	English	Sarcasm	Not mentioned	
Zampieri, et al. [57]	Twitter	One	English	Offense	Manual	Figure Eight
Zhang et al. [59]	Twitter	Seven	English	Irony	Manual and automated	Experts and self-annotating using hashtags
Fortunatus et al. [4]	Facebook	One	English	Cyberbullying	Manual	Experts
González et al. [65]	Twitter	Two	English and Spanish	Irony	Followed Van Hee et al. [62] and Ortega-Bueno et al.'s [42] approaches	
Iwendi et al. [2]	Wikipedia	One	English	Cyberbullying	Followed Wulczyn et al.'s [55] approach	
Paul and Saha [35]	Formspring, Twitter, and Wikipedia	Three	English	Cyberbullying	Followed Waseem and Hovy [47], Wulczyn et al. [55], and Reynolds et al.'s [50] approaches	
Potamias et al. [66]	Twitter and Reddit	Four	English	Irony and sarcasm	Not mentioned	
Rezvani et al. [67]	Instagram and Twitter	Two	English	Cyberbullying	Followed Hosseinmardi et al. [51] and Bamman and Smith's [68] approaches	
Tripathy et al. [37]	Twitter	One	English	Cyberbullying	Followed Davidson et al.'s [53] approaches	

**Table 3:** Summary of preprocessing steps.

Research	Preprocessing techniques				Preprocessing steps
	Replace @, URL, #, RT	Tokenization	Stop word/punctuation removal	Other techniques	
Potha and Maragoudakis [43]	✓	✓	✓	Converted letters to lower case	They applied tokenization based on the space character, stop word removal, and a case transformation as preprocessing steps
Ptacek et al. [17]	✓	✓	✓	Stemming	They replaced user, URL, and hashtag in tweets by “user,” “link,” and “hashtag.” They removed retweets starting with “RT” and removed diacritics from all Czech tweets. They also used tokenization, POS tagging, stem, stop word removal, and phonetics.
Rajadesingan et al. [61]	✓	N/A	N/A	Removed tweets with three words or less	They removed non-English tweets, retweets, tweets that contained mentions and URLs, and tweets containing three words or less such as yeah, right, and so on as they found that these words were very noisy.
Capua et al. [21]	N/A	N/A	✓	Stemming	They applied stop word and punctuation removal and stemming.
Van Hee et al. [38]	✓	✓	N/A	Replaced emojis, POS, and lemmatization	They replaced all emojis with their name or description, normalized hyperlinks, and retweets to https://someurl and @someuser, respectively. They also applied tokenization, PoS tagging, lemmatization, and named entity recognition.
Nand et al. [18]	✓	N/A	N/A	Replaced word abbreviation	They used Nand et al. techniques for eliminating noise such as word variations e.g., replacing tmro and 2moro by tomorrow, multi-word abbreviation, e.g., replacing lol with laugh out loud, slangs, e.g., replacing gonna by going to, and removed duplicates, retweets, @usernames, #hashtags, hyperlinks.
Zhang et al. [26]	N/A	N/A	N/A	Removed sarcasm hashtags	They removed the hashtags #sarcasm and #not from the tweets and assigning to them the sarcasm output tags for training and evaluation.
Zhao and Mao [28]	✓	✓	✓	N/A	They followed Xu et al.'s preprocessing steps for Twitter dataset using only tokenization with replacing special characters such as mentions @ and URLs with tokens “@USERNAME” and “HTTPLINK,” respectively. They included hashtags and emoticons as tokens. For MySpace dataset, their focus was on content-based and the preprocessing for text only using tokenization and deletion of punctuation and special characters.
Bharti et al. [20]	✓	N/A	N/A	Converted letters to lower case	They removed retweet, hashtags, URL, and @username and converted letters to lower cases.
Felbo et al. [83]	✓	N/A	N/A	Removed characters repeated more than twice	They used English tweets without URLs, removed characters repeated more than twice, and replaced mentions and numbers with special tokens.
Romsaiyud et al. [22]	N/A	N/A	N/A	Removed non-printable and special characters and duplicate words	They preprocessed their datasets using a method to remove non-printable and special characters and duplicate words.
Mishra et al. [46]	N/A	N/A	✓	Converted letters to lower case	They changed all letters to lower case and removed stop words to normalize the data.
Rosa et al. [25]	✓	N/A	N/A	Removed characters repeated in the words more than twice	They removed “Q” and “A” markers, “html” tags, and repeated characters in the words more than twice.
Cai et al. [32]	N/A	N/A	N/A	Separated words, emoticons, and hashtags	They cleaned up their dataset by rejecting tweets using the words sarcasm, sarcastic, irony, and ironic as regular words, tweets containing URL's, and tweets with words that frequently co-occur with sarcastic tweets. In the preprocessing phase, mentions of (@user) were replaced with <user>, and then they used the NLTK toolkit to separate words, emoticons, and hashtags.
Drishya et al. [63]	N/A	N/A	✓	Stemming	They cleaned their dataset by conducting stemming and removing stop words.
Samghabadi et al. [54]	✓	N/A	N/A	Converted letters to lower case and padding	They changed all letters to lower case and replaced all of the links and user mentions with the words “url” and “@username” respectively, truncated the posts to 200 tokens, and left-pad the shorter sequence with zeros.
Singh et al. [74]	✓	✓	✓	Converted letters to lower case and removed repeated words	They used tokenization, changed all letters to lower case, and removed stop words, numbers, URLs, consecutive repeated words, user mentions, and expand hashtags.
Subramanian et al. [33]	✓	N/A	✓	N/A	They preprocessed their datasets using removal of hyperlinks, special characters, hashtags, retweets, etc.
Fortunatus et al. [4]	N/A	N/A	✓	Replaced emojis and emoticons with their Unicode, normalization, and POS	They started their cleaning process with separating emojis and emoticons from plain text utilizing emoji Unicode representation from emoji sentiment ranking and emoticon Unicode from emoticon lexicon. The other preprocessing steps they used included punctuation removal and text normalization which included five steps: pronoun spelling resolution, slang resolution, laughter text resolution, elongated character reduction, and similar word replacement. After the normalization step, they used NLTK's POS tagger to perform part-of-speech tagging. The last preprocessing step was stop removal which was done after POS tagging to ensure that the POS tagger can work as effective as possible; otherwise, some words might already be removed and the POS tag will not be correct because the sentence is no longer grammatically sensible.
González et al. [65]	✓	✓	N/A	Removed characters repeated in the words more than twice	They applied a case-folding process for all the tweets, used TokTokTokenizer from NLTK to tokenize the tweets, replaced user mentions, hashtags, and URLs by the token user, hashtag, and URL, respectively, and removed repeated characters in the words more than twice.
Gupta et al. [84]	N/A	N/A	✓	N/A	They used stop word removal algorithm and filtration technique as preprocessing step to remove stop words from the dataset.
Iwendi et al. [2]	N/A	✓	✓	Stemming, lemmatization, and converted letters to lower case	They started the preprocessing by removing punctuation and non-letter characters and changed all letters to lower case. Then, they tokenized the text by separating it into smaller tokens that may include words, numbers, and punctuation marks. Next, they used stemming to refer each token to its root such as removing plurals and verb tense, e.g., converting the words “running,” “ran,” and “runner” to “run.” After stemming, they used lemmatization. The Twitter dataset that was used by Paul and Saha was preprocessed by its author by normalizing the data through removing stop words, special markers such as “RT” (retweet) and screen names, and punctuation.
Potamias et al. [66]	N/A	N/A	N/A	Converted letters to lower case	They used only decapitalization as a preprocessing step.
